# Death of Dr. Charles H. Wilcox

**Published:** 1862-12

**Authors:** 


### EDITORIAL DEPARTMENT.

### DEATH OF DR. CHARLES H. WILCOX.

In our last issue we noticed the sickness and we are now pained to announce the death of a most worthy and estimable physician of our City, Dr. Charles H. Wilcox.

At a meeting of the Buffalo Medical Association and Erie County Medical Society, held Nov. 7th, on the occasion of his death, his friends made extended remarks, giving history of his early life, professional standing, and faithful service to his country,

While we regret our inability to publish in full all the remarks made upon that occasion, we shall take the liberty to extract from the addresses of two or three of his most intimate friends, enough to show our readers the prominent characteristics of his life and the feeling of sorrow and sadness which pervaded the profession at the announcement of his death.

Prof. White, as President of the meeting, after announcing his death, gave a brief history of his life, while a student of medicine in his office and member of his family, and also of his professional life and services, from which we extract the following:

"In the spring of 1861, when Buffalo sent her first regiment to the war, he was induced by his patriotic impulses, urged as he was by myself and others, to accept the appointment of surgeon, and immediately assumed its duties. From that time forward, whether in camp or in battle, he has been untiring in his labors for the welfare of those under his charge.

Indeed his camp was held up by those in authority as a model in all its hygienic arrangements. Unsparing of himself, his first care was to see that all his men were properly provided with every means for the preservation of health, or with everything which could contribute to their comfort or restoration of the sick. He soon secured the confidence of the medical authorities, which brought increased labors as well as honors. Without solicitation he was promoted from Regimental to Brigadier Surgeon, in which more extended sphere the concurrent testimony of all, from general to private is, that his duties were so faithfully and satisfactorily discharged as to command universal respect, confidence and admiration. Finally, after the battle of Antietam, when already exhausted with care of the wounded of his regiment, he was charged with the organization of a new hospital, which should hold five hundred beds. Never shrinking from any labor which might be demanded of him, he resolutely entered upon the undertaking. To those who knew his determined spirit, I need scarcely add it was accomplished, although his own life has paid the penalty of his unceasing toil and care. Exhausted, he was attacked with the disease which has found so many victims among our Northern heroes, and after a fortnight of severe suffering there, he was enabled to return and die in the bosom of his family and surrounded by his friends. Long will his memory be cherished by those who have been relieved by his ministrations, whether in civil or military practice, and by the many friends who knew and appreciated his large hearted and generous spirit. To his family, to the Regiment to whom he has been a father, and to the sick poor, his loss is irreparable.

Dr. Wilcox possessed a mind of great strength and clearness, and those who knew him best respected him most, his good sense and sound judgment.

His language sometimes failed adequately to convey a just idea of sound conclusions, at which he had arrived by a severe analysis of facts. This was the misfortune of a deficient early classical education, against which he was ever compelled to struggle, and which to the superficial observer, might diminish the weight of his opinions.

In all associate efforts for professional improvement and intercourse, he was prompt in his attendance, unselfish in his efforts to promote their success, and generous in his contributions. His conduct with his professional brethern, wherever he met them, was eminently distinguished by candor and liberality, and the total absence of professional trick. He never attempted to make himself of importance, but was ever ready to give the strongest commendation to the course of conduct pursued by others when he thought it judicious, and ever manifested an utter detestation of all those professional arts by which the favor of the public is sometimes too successfully propitiated. Never wanting in professional zeal or integrity, warm and steadfast in his attachments, of a generous disposition—bordering on profusion, instinctively shrinking from any proceeding liable to the slightest imputation of meanness, selfishness or duplicity, he was a noble competitor and an unflinching friend."

We quote also the closing remarks of Prof. Rochester:

"On an occasion like the present, words fail to express the emotions of the heart. Charles H. Wilcox is dead. When this sad event was announced, although long anticipated and dreaded, it come as a shock upon all, and deep grief welled up in the heart and burst from the lip of each one who knew him. He was one of my earliest and truest friends in Buffalo, honest tender and affectionate, a rough diamond, uncut and unpolished, but, a gem of the purest water. He died as we should all wish to die, from disease induced by the earnest and faithful practice of his profession. He died, not as the warrior, in his cloak of martial glory, with Fame ready to sound his praises over the land, but quietly, at home, surrounded by his family and friends, remote from the scenes of his labors and his dangers. His name will not be bruited far and wide, but it will be remembered and cherished through life, by those who were fortunate enough to have been worthy of his friendship.

I move, Mr. President, that a committee be appointed to draft resolutions expressive of our respect and of our grief."

Dr. T. T. Lockwood closed his remarks by the following truthful and graphic account of the last efforts of his life:

"During and after the battle of Antietam, he was unwearied, both by night and day, in his exertions to alleviate the sufferings of the wounded.—He furnished a model to his brother surgeons of tenderness, skill and unflagging faithfulness. It is known that his death is attributed to over exertion in his almost superhuman efforts to alleviate the horrors of that last dreadful field.

His regiment will mourn for him with a sorrow sharpened by the consciousness that its loss can scarcely be repaired. Our city may well mourn, for it has not many citizens who are more useful, more respected, more widely beloved than was Dr. Wilcox. The tribute of respect which we pay to his memory is interwoven with the more eloquent testimony that comes up from the bloody fields of Maryland and Virginia. Soldier and civilian unite with us in deploring the death of a brave, good man, an able physician, a true christian hero."

After remarks by Drs. Eastman, Gay, Storck, Miner and others, a committee, consisting of Drs. Rochester, Eastman and Lockwood, presented the following resolutions which were unanimously adopted.

Our friend, associate, and professional brother, DR. CHARLES H. WILCOX, is *dead*—self-immolated upon the altar of our country, and a victim to his untiring and self-sacrificing devotion to the sick and wounded champions of our liberty and national unity. We, the physicians of Buffalo, and the members of the City and County Medical Societies, are convened, on account of this afflictive event, to do honor to the memory of one who, by his honesty, integrity and earnestness of purpose, was endeared to one and all of us, and, as a record and testimonial of our esteem and of our loss, it is by us

*Resolved*, That, as medical men and members of the aforesaid medical Societies, we have lost one of our most valued and cherished associates, and that, as Americans and citizens, we mourn the death of one of the purest of patriots and one of the best of men.

*Resolved*, That we tender to his family our warmest sympathy in this their deep bereavement.

*Resolved*, That we will employ our best endeavors to secure a suitable maintenance to his widow and orphans, left desolate and dependent by his devoted death.

*Resolved*, That we will attend the funeral of Dr. Wilcox. and wear the customary badge of mourning.

*Resolved*, That a copy of this preamble and resolutions be sent to the family of the deceased, and to the *Buffalo Medical Journal*, and to the City papers for publication.

---

The Old Medical College, in Woodstock, Vermont, has been sold at auction. It brought, with the appurtenances, the sum of seven hundred and fifty dollars.

